# Epigenetic and Transcriptional Regulation in the Induction, Maintenance, Heterogeneity, and Recall-Response of Effector and Memory Th2 Cells

**DOI:** 10.3389/fimmu.2018.02929

**Published:** 2018-12-12

**Authors:** Atsushi Onodera, Kota Kokubo, Toshinori Nakayama

**Affiliations:** ^1^Department of Immunology, Graduate School of Medicine, Chiba University, Chiba, Japan; ^2^Institue for Global Prominent Research, Chiba University, Chiba, Japan

**Keywords:** polycomb and trithorax, airway infiammation, pathogenic Th2 (Tpath2) cells, GATA3, allergic disease

## Abstract

Antigen-primed T cells respond to restimulation much faster than naïve T cells and form the cellular basis of immunological memory. The formation of memory Th2 cells starts when naïve CD4 T cells are transformed into effector Th2 cells and is completed after antigen clearance and a long-term resting phase accompanied by epigenetic changes in the Th2 signature genes. Memory Th2 cells maintain their functions and acquired heterogeneity through epigenetic machinery, on which the recall-response of memory Th2 cells is also dependent. We provide an overview of the epigenetics in the whole Th2 cell cycle, mainly focusing on two different histone lysine methyltransferase complexes: the Polycomb and Trithorax groups. We finally discuss the pathophysiology and potential therapeutic strategies for the treatment of Th2-mediated inflammatory diseases in mice and humans.

## Introduction

T cells experience several events before transforming into memory T cells: antigen priming, differentiation into certain functional distinct subsets, migration to inflammatory sites, exertion of the effector functions, and a long-term resting phase. Some of these events are unnecessary or may even be inadvisable for memory T cell formation. Antigen priming, however, is definitely essential for the formation of the immunological memory (1–4). During antigen priming, T cell receptor (TCR) signals induce epigenetic changes of the genes encoding lineage-specifying transcription factors and lineage-specific cytokines collaborating with signals from costimulatory molecules and cytokine receptors (5). After undergoing the above-described cellular events, T cells finally become memory T cells, in which the genes responsible for a rapid response to the same antigen are epigenetically poised for transcription. In this review, which focuses on Th2 cells, we discuss the epigenetic regulatory mechanisms underlying T cell-mediated immune responses beginning from the priming of naïve T cells and ending with the recall-response of memory T cells.

**Figure 1:**
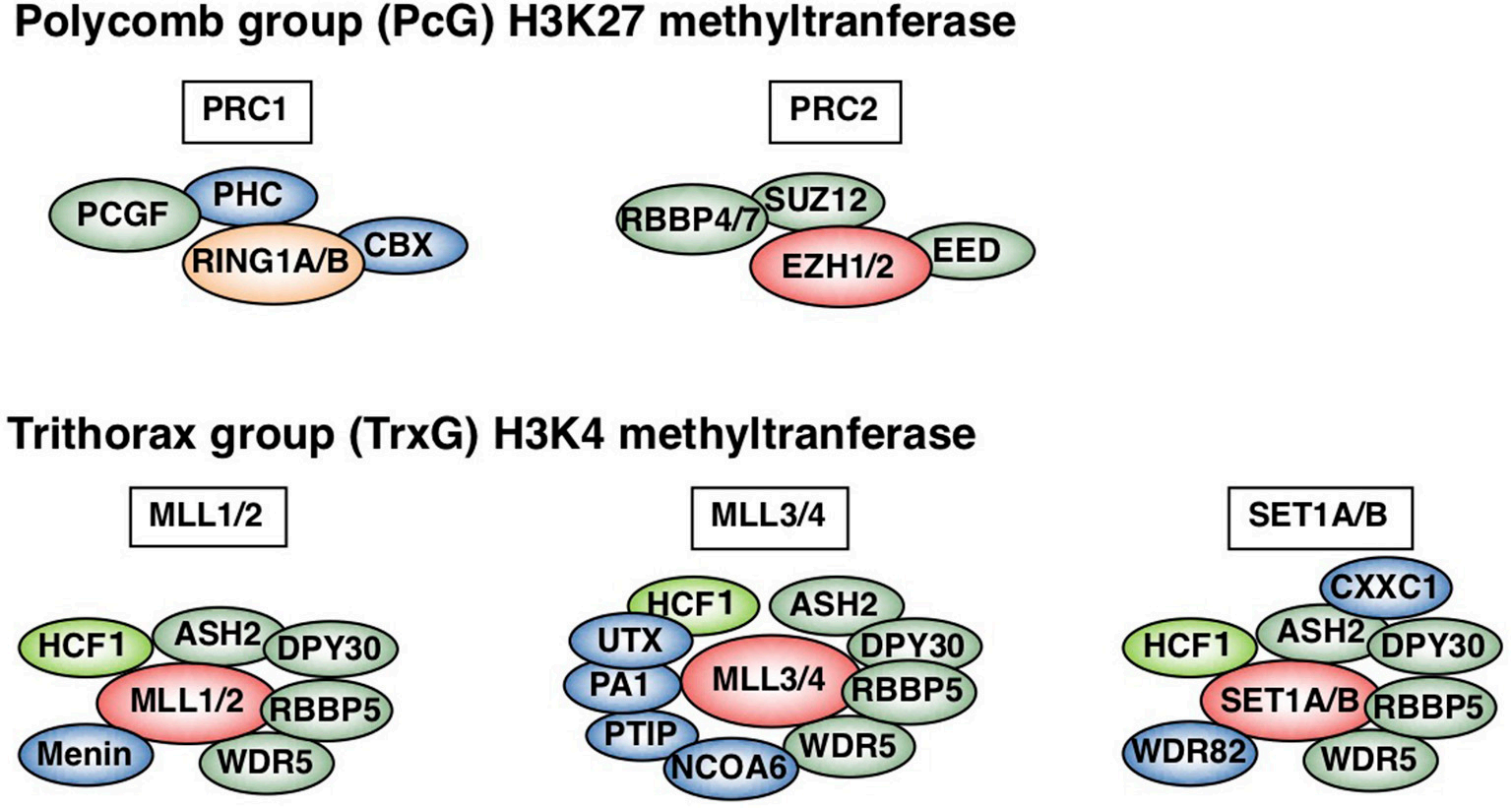
Polycomb (PcG) and Trithorax (TrxG) complexes in mammals. Two basic types of Polycomb repressive complex 1 (PRC1) and PRC2 are shown (upper). Canonical PRC1 consists of four core subunits: RING1A/B, PCGF, CBX, and PHC (1, 15, 16). PCGF and RING1A/B, which ubiquitinate H2AK119, also compose non-canonical PRC1 (15). PCGF4 is also known as Bmi1. PRC2 consists of four core subunits: EZH1/2, EED, SUZ12, and RBBP4/7. The SET domain of EZH1/2 is responsible for PRC2 methylase activity. In contrast, mammalian cells have six H3K4 methylases: MLL1-4, SET1A, and SET1B (lower) (1, 15–17). All of these complexes share ASH2L, RBBP5, DPY30, WDR5, and HCF1, which is a substoichiometric component that is absent in some branches of the TrxG complexes (green) (17). Menin is a unique subunit of MLL1/2 complexes (blue). MLL3/4 complexes are uniquely associated with PTIP, PA1, UTX, and NCOA6, while SET1A/B complexes are specifically associated with WDR82 and CXXC1 (shown in blue). This figure was reproduced with permission provided by Annual Reviews copyright transfer agreement [originally published by Nakayama et al. (1)].

In contrast to innate immunity, acquired immunity recognizes non-self-peptide antigens through TCRs on naïve CD4 T cells, resulting in the functional differentiation of effector helper T (Th) cell subsets, including Th1, Th2, and Th17 cells (6). Each subset has its “working range” in immune response. For example, Th1 cells organize CD8 T cell-mediated cellular immunity against intracellular bacteria and viruses by producing IFNγ. However, Th1 responses are often associated with tissue-specific autoimmune diseases, including type 1 diabetes (7). Th2 cells produce IL-4, IL-5, and IL-13 (so-called Th2 cytokines) and play a role in immunity against extracellular parasites (1). Th2 cells also cause allergic diseases, including asthma, rhinitis, and atopic dermatitis. Th17 cells secrete IL-17 and are crucial for immunity against fungi; they are also involved in the pathogenesis of inflammatory bowel disease in collaboration with Th1 cells (8). The differentiation of each Th subset accompanies epigenetic changes in its specific genes (9). Thus, regulatory molecules in the epigenetic changes have received significant attention in the field of immunology. Histone modifications, DNA methylation, and non-coding RNA transcripts, such as microRNAs and long non-coding RNAs (lncRNAs), are now recognized as important epigenetic regulators (10–12). Various post-translational modifications of histone tails, which are tightly associated with gene expression, have been identified. The methylation of histone H3K27 is considered to be important for gene silencing and is catalyzed by Polycomb group (PcG) proteins (1, 13–15). PcG complexes were originally identified in Drosophila and are categorized into two basic types: Polycomb repressive complex (PRC) 1 and 2 (Figure 1). Enhancer of Zeste (EZH) 1 and 2, which methylate H3K27, are active subunits of PRC2. PRC1 recognizes and binds to H3K27 methylation and represses the target gene expression in collaboration with PRC2. Another subunit of PRC1, ring finger protein (RING1), possesses ubiquitin ligase activity for histone H2AK119. In contrast to H3K27 methylation—which is mediated by PcG proteins—H3K4 methylation, which is catalyzed by Trithorax (TrxG) proteins, is associated with a chromatin structure that permits transcription (Figure 1). In mammals, six H3K4 methylases have been identified and classified into three groups (15–17). The first group consists of mixed lineage leukemia (MLL)-1/2 and a specific component, Menin, which is encoded by the *MEN1* gene in humans, the mutation of which is often associated with multiple endocrine neoplasia type 1 (MEN1). The second group contains MLL-3/4 and H3K27 demethylase, UTX (ubiquitously transcribed tetratricopeptide repeat, X chromosome). The translocation or mutation of the genes encoding MLL proteins are frequently found in leukemia patients, indicating that appropriate control of the MLL functions is important for the homeostasis of hematopoiesis. The third group of H3K4 methylase complex is composed of SET1A/B and the unique subunit WDR82. TrxG proteins can both upregulate the expression of the target gene and keep it active, depending on their association partners or the epigenetic signatures of the target genes (18). The present review mainly focuses on the PcG- and TrxG-mediated epigenetic regulation of effector and memory Th2 cells, which have dual aspects in the immune system: protective and pathogenic.

## Epigenetic Regulation in the Induction of Th2 Cell Differentiation

### STAT6 Is Activated by IL-4 Signaling and Induces Epigenetic Changes of the *Gata3* Gene

**Figure 2:**
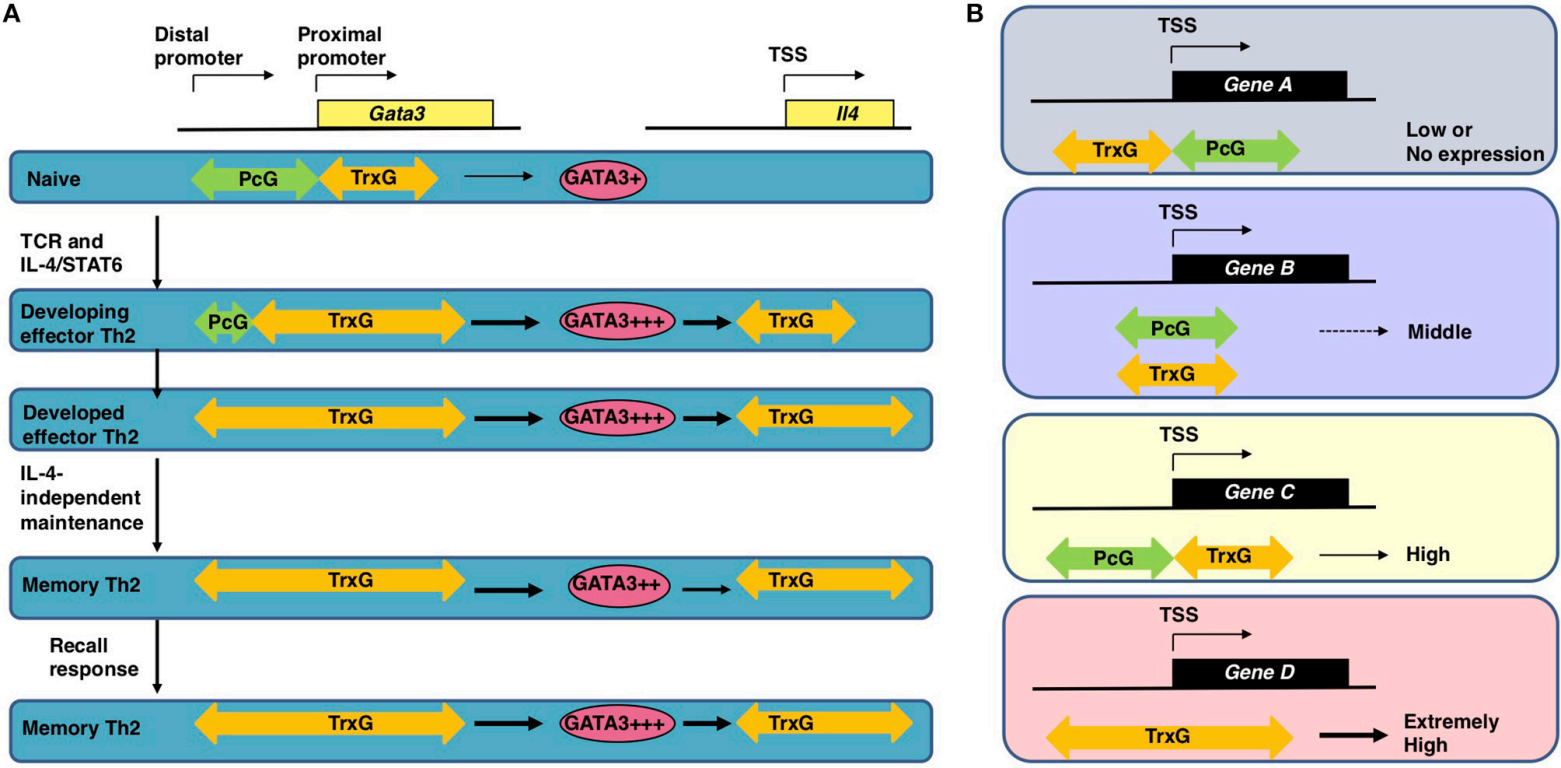
**(A)** The epigenetic regulation of the induction, maintenance, and recall-response of effector and memory Th2 cells (1, 22). In naïve CD4 T cells, which express a moderate level of *Gata3* mRNA, the PcG proteins bind to the region between the distal and proximal promoter of the *Gata3* gene. After receiving signals via the T cell receptors (TCRs) in the presence of IL-4, activated STAT6 proteins bind to the *Gata3* gene locus, resulting in disassociation of the PcG complex and spreading of the TrxG complex binding to the region between the distal and proximal promoters. Extremely high *Gata3* mRNA expression levels are achieved “in an IL-4/STAT6-dependent but TrxG-independent manner” in developing Th2 cells (1, 22). During Th2 cell differentiation TrxG is also recruited to the *Il4* gene locus and induces H3K4 methylation and H3K9 acetylation in collaboration with GATA3 proteins. Once Th2 cells are differentiated, the TrxG protein binding is observed from the proximal promoter to intron 3 of the *Gata3* gene accompanied by a broad range of H3K9ac and H3K4me3. This TrxG binding pattern may be preserved during the process of generating memory Th2 cells from effector Th2 cells. The TrxG complex bound to the *Gata3* locus can maintain the strong expression of *Gata3* in memory Th2 cells where the transcription of *Gata3* is regulated “in an IL-4/STAT6-independent but TrxG-dependent manner” (1, 22). Note that in memory Th2 cells, the protein expression of GATA3 is slightly decreased in comparison to effector Th2 cells because GATA3 proteins are unstable in resting conditions. When memory Th2 cells reencounter their cognate antigens and a recall-response is induced, the GATA3 protein expression is immediately upregulated to produce large amounts of Th2 cytokines. TrxG proteins bound to the *Il4* gene also play an important role in the expression of IL-4 in memory Th2 cells. This figure was reproduced according to the permissions policy of Rockefeller University Press Journals © 2010 Onodera et al. **(B)** The spatial interplay between Polycomb (PcG) and Trithorax (TrxG) proteins. In CD4 T cells, the binding position of PcG and TrxG proteins relative to the transcription start site (TSS) of a gene is connected to its expression (1, 23). PcG and TrxG proteins show an exclusive binding pattern at the TSS of some genes: PcG is observed to bind downstream of the TSS while TrxG binds upstream of the TSS in genes with lower transcription levels (Gene A); in contrast, PcG is observed to bind upstream of the TSS while TrxG binds downstream of the TSS in genes with higher transcription levels (Gene C). When TrxG proteins bind throughout a gene, the gene shows an extremely high transcription level (Gene D). When PcG and TrxG proteins are bound in a similar position relative to the TSS, the transcription level of this gene is expected to be moderate (Gene B). This figure was reproduced according to the permissions policy of ASM Journals Copyright © Onodera et al. (23).

Antigen recognition via TCR is an essential event for naïve CD4 T cells to initiate clonal expansion and differentiation into effector Th cell subsets, including Th2 cells. The TCR signaling pathway is known to turn on the activation switch of naïve CD4 T cells, whereas cytokines and their receptor signaling pathways direct the differentiation of naïve CD4 T cells toward each subset. Th2 differentiation is induced by IL-4 and its receptor signaling cascade, which finally phosphorylates STAT6. Phosphorylated STAT6 forms a dimer, moves into the nucleus, binds to the target genes, and controls their expression (19, 20). The most important target of STAT6 is the *Gata3* gene, which encodes a transcription factor, GATA3, the element responsible for the chromatin remodeling of Th2 cytokine gene loci. Actually, the direct binding of STAT6 is determined within the *Gata3* gene locus by both ChIP-seq and conventional ChIP assays (21, 22). IL-4 fails to upregulate the expression of *Gata3* without STAT6. Consequently, very few IL-4-producing Th2 cells can be generated from STAT6-deficient naïve CD4 T cells, even when cultured under Th2-inducing conditions. STAT6 also plays a role in the epigenetic regulation of the *Gata3* gene during Th2 cell differentiation (Figure 2). The *Gata3* gene is known to have two promoters: a proximal promoter and a distal promoter, the latter of which is located approximately 10 kilobases upstream of the transcription start site (TSS) (24). *Gata3* transcription is mainly dependent on the proximal promoter in both naïve CD4 T and Th2 cells, although qPCR (quantitative polymerase chain reaction) detected a small amount of transcripts driven by the distal promoter in Th2 cells (22, 25). A dramatic change in the epigenetic marks is observed between the distal and proximal promoters during Th2 cell differentiation. In naïve CD4 T cells, the binding of PcG proteins is detected in these regions. In contrast, TrxG proteins bind to the proximal promoter and its downstream region. Thus, the proximal promoter forms a boundary between the PcG-binding and TrxG-binding regions. During Th2 cell differentiation, PcG proteins disassociate from the region between the distal and proximal promoters, and the binding of TrxG proteins spreads into this region. Basically, histone modification patterns behave in a similar way. H3K27 is highly methylated in the region between the distal and proximal promoters in naïve CD4 T cells and demethylated during Th2 differentiation. H3K4me3, which is found at the proximal promoter and its downstream region in naïve CD4 T cells, spreads upstream. Thus, the exchange of PcG and TrxG at the region between the distal and proximal promoters of the *Gata3* gene is induced by STAT6 and defines the Th2 cell identity.

### Spatial Interplay Between the Polycomb and Trithorax Complexes

The *Gata3* gene is co-occupied by PcG and TrxG proteins and shows bivalency, with both H3K27me3 and H3K4me3 being present at the same time in naïve CD4 T cells (26). The functions of these bivalent genes were originally analyzed in embryonic stem (ES) cells and are poorly understood in T cells (27). Furthermore, there are few reports on genes co-occupied by PcG and TrxG proteins. We therefore analyzed the features of the co-occupied genes in both ES and T cells. A substantial number of the co-occupied genes are found in ES cells, whereas only a few genes are co-occupied by PcG and TrxG proteins in T cells (23). The binding levels of PcG proteins and those of TrxG proteins are reciprocally correlated in both cell types. In this study, we also identified two binding patterns: “PcG bound upstream and TrxG bound downstream of the TSS,” a pattern that was frequently observed in strongly expressed genes in T cells; and “PcG bound downstream and TrxG bound upstream of the TSS,” a pattern that was frequently observed in weakly expressed genes in T cells (Figure 2) (23). Interestingly, the former gene group includes *Nfatc1, Fli1*, and *Gfi1*, which are important for the development and function of T cells (23). Thus, spatial interplay between the PcG and TrxG proteins may be a novel mechanism regulating the bivalent genes co-occupied by these two complexes. It has been proposed that PcG proteins maintain the *Gata3* expression at “an appropriate level in naïve CD4 T cells” based on observations in *Ezh*2 knockout mice: CD4 T cells that lack *Ezh2* showed enhanced sensitivity to IL-4, increased *Gata3* expression, and Th2 cytokine hyper-production (1, 28). In contrast to the *Gata3* gene locus, the Ezh2 binding levels at Th2 cytokine gene loci were very low, suggesting that the Th2 cytokine expression is controlled though Ezh2-dependent repression of the *Gata3* gene.

### The GATA3-Dependent Epigenetic Regulation of Th2 Cytokines and Other Th2 Signature Genes

The GATA family transcription factors (GATA1-6) recognize the consensus DNA sequence WGATAR via one or two C2-C2-type zinc-finger motifs (29–31). Based on their expression patterns in the body, GATA1-3 are classified as hematopoietic factors, whereas GATA4-6 are recognized as endodermal factors. In the immune system, GATA3 is predominantly expressed in T cells and innate lymphoid cells (ILCs), including natural killer (NK) cells (32). Regarding T cells, GATA3 exercises important functions to go through the β-selection checkpoint during the CD4 versus CD8 lineage choice and it is indispensable for the development and maturation of CD4 single-positive (SP) thymocytes (33–36). One of the important roles of GATA3 in the thymus is regulating the expression of Th-POK, which is an essential transcription factor for CD4-SP T cell development (37, 38). Another role of GATA3 in the thymus is controlling a set of genes encoding TCR components, including *Cd3d* and *Cd3e* (37). Deletion of the *Gata3* gene results in the decreased expression of CD3 in double-positive (DP) T cells, indicating that GATA3-dependent TCR signal strength play an important role in thymocyte development (37). GATA3 is continuously expressed at a basal level in peripheral naïve CD4 T cells, until IL-4/IL-4 receptor signaling activates STAT6 and induces the upregulation of the mRNA expression of *Gata3* (39). The high-level expression of GATA3 has been proposed to induce histone H3K4 methylation and H3K9 acetylation in so-called Th2 cytokine gene loci, which include the *Il4, Il5*, and *Il13* genes, during development of Th2 cells (40). These epigenetic changes play important roles in the formation of the accessible regions for transcription factor binding, which can be detected as DNase I hypersensitive (HS) sites. A recently developed technique, assay for transposase-accessible chromatin sequencing (ATAC-seq), has proven useful for analyzing these highly accessible regions (41). The enforced expression of GATA3 by a retroviral vector induces IL-4-producing Th2 cell differentiation, even if naïve CD4 T cells are cultured under Th1-inducing conditions, indicating that GATA3 is the necessary and sufficient master transcription factor for Th2 cell differentiation (39, 42, 43). The retroviral exogenous expression of GATA3 is shown to upregulate the endogenous GATA3 expression, and correspondingly, a single peak of GATA3 binding is detected in the *Gata3* gene and is located close to one of the STAT6 binding sites (44, 45). In addition, the GATA3 protein expression levels are tightly regulated by various posttranscriptional mechanisms in Th2 cells (46–48). “A conserved YxKxHxxxRP motif” in the C-terminal zinc finger domain of GATA3 protein has been shown to be critical for binding to DNA, inducing chromatin remodeling at Th2 cytokine gene loci, and exerting transcription factor activity (49). GATA3 is also known to be associated with some cofactors and to organize functionally distinct complexes (1). Fli1, an Ets family protein, is shown to colocalize with GATA3 and facilitate GATA3 functions (37). Chromodomain helicase DNA-binding protein 4 (Chd4) is proposed to interact with GATA3 and p300 and be involved in GATA3-dependent transcriptional activation (50). In contrast, Chd4 is also involved in GATA3-dependent gene silencing when interacting with GATA3 and nucleosome remodeling histone deacetylase (NuRD) (50). A recent study reported an interesting binding partner of GATA3, Bcl11b, which plays an important role in limiting the Th2-related gene expression and suppressing the non-Th2 gene expression (51). It has been reported that several cis-regulatory elements (also known as locus control regions) at Th2 cytokine gene loci are also bound by GATA3. These regulatory elements include the conserved GATA response element (CGRE), the conserved non-coding sequence (CNS)-1, CNS-2, hypersensitive site HSVa, and HSII within the *Il4* gene (52–56). CGRE, which was originally identified in 2002 as a region containing four consensus GATA-binding sequences, overlaps with the previously identified HSI. This region is located 1.6 kilobases upstream of the TSS of the *Il13* gene (57). Correspondingly, strong GATA3 binding signals have been detected in the CGRE (37, 45, 58). Interestingly, the CGRE forms a boundary between hyper- and hypo-acetylated regions. This fact implies that GATA3 primarily binds to the CGRE and secondarily spreads histone hyperacetylation toward the 3′-end of the *Il13* gene (52). Indeed, the association of GATA3 with histone acetyltransferases CBP, p300, and RNA polymerase II is observed in this region (57, 59). Thus, the CGRE region may function as a regulatory element for chromatin remodeling at the *Il13* locus and subsequent mRNA expression of *Il13*. Notably, when Th2 cells are generated from naïve CD4 T cells of CGRE-deficient mice, the diminished IL-13 production but normal IL-4 or IL-5 production is observed, suggesting that a compensatory mechanism underlies the IL-4 and IL-5 production in the absence of this region (60). Genome-wide, GATA3 has been shown to regulate H3K4 methylation in enhancers, including these locus control regions; H3K4me2 levels are decreased in GATA3-deficient Th2 cells at non-promoter GATA3 biding sites (37).

In addition to epigenetic regulation, GATA3 is known to act as a transcription factor for the *Il5* and *Il13* genes: GATA3 directly binds to the promoters of these cytokine genes and induces transcription upon TCR restimulation (61–63). In fact, the decreased expression of *Il5* and *Il13* was observed in differentiated effector Th2 cells in which the *Gata3* gene was knocked down by siRNA just before TCR restimulation. Furthermore, other Th2 signature genes are transcriptionally regulated by GATA3 in effector Th2 cells (52). The expression of approximately half of the Th2-specific genes (16 out of 31) in effector Th2 cells was significantly reduced by *Gata3* siRNA knockdown; the *Tube1* gene was the only gene for which the expression was significantly increased, indicating that one of the major roles of GATA3 is the transcriptional activation of target genes (52, 58). In contrast, the transcription of other Th2-specific genes is not affected by *Gata3* siRNA knockdown. This fact implies that GATA3 is a master regulator for Th2 cytokine expression but not for all Th2 signature genes. A similar observation was reported in a study in which the expression of approximately half of a different set of Th2-specific genes (44 out of 90) was decreased in Th2 cells by *Gata3* knockout (37). The authors of that report noticed some interesting rules regarding GATA3-dependent transcriptional regulation. First, the genes positively regulated by GATA3 were found in the strongly expressed gene group while the genes negatively regulated by GATA3 were found in the weakly expressed gene group (37). Second, the authors argue that genes with higher numbers of GATA3 peaks tend to be affected by *Gata3* knockout. This appears to be true for genes both positively and negatively regulated by GATA3. Taken together, these findings suggest that the Th2-specific upregulation of GATA3 epigenetically and transcriptionally induces a set of Th2 signature genes as well as represses another set of genes that specifies other Th subsets. Approximately half of the Th2-specific genes are affected by *Gata3* knockdown or knockout, leaving the other half of Th2-specific genes intact.

### Epigenetic Mechanisms That Are Shared Between Th2 Cells and Other Conventional or Unconventional T Cells

DNA methylation is generally observed at cytosine of the CpG sequences in the genome. Dnmt1 is reported to be a maintenance enzyme responsible for converting hemi-methylated CpG into symmetrically methylated CpG after DNA replication (64). Genetic deletion of the *Dnmt1* gene results in the increased expression of both IL-4 and IFNγ in Th1 and Th2 cells and under unpolarizing conditions (65–67). Thus, Dnmt1-mediated gene silencing is important for preventing the excess production of these cytokines and modulating the proper differentiation of Th1 and Th2 cells. Th2 cells also share several molecular mechanisms with Th2-like unconventional T cells, including NKT2 cells (68, 69). In the absence of *Gata3*, a significant reduction in IL-4 production was observed in iNKT cells, indicating that GATA3 plays a crucial role in NKT2 cell development in the thymus (70). In addition, growth factor-independent-1 (Gfi-1) regulates the GATA3 protein expression in Th2 cells and iNKT cells. Gfi-1 knockout results in decreased IL-5 production and increased IFNγ production in Th2 cells, whereas both IFNγ-producing NKT1 and IL-4-producing NKT2 cells are abrogated in the absence of Gfi-1 in the thymus (46, 71). As described above, Th2 cell differentiation is considered to be controlled by both Th2-specific mechanisms and general epigenetic machineries shared with conventional and unconventional T cells.

## Epigenetic Regulation in the Maintenance of the Memory Th2 Cell Functions

### Maintenance of the Memory Th2 Cell Function Depends on by Trithorax Molecules, MLL1, and Menin

Antigen-primed Th cells migrate to inflammatory sites in peripheral tissues and produce large amounts of effector cytokines when they reencounter their cognate antigens in order to eliminate these antigens. After antigen clearance, it is thought that most of these antigen-reactive effector Th cells die due to apoptosis in the contraction phase. However, some of the effector Th cells survive during the contraction phase, resulting in the generation of memory Th cells that can rapidly respond in cases of secondary antigen exposure (72). In general, CD4 T cells are thought to start acquiring the epigenetic signatures of memory Th cells from priming, which is almost established in differentiated Th subsets (5). The TrxG-binding pattern of the *Gata3* gene, which is established during Th2 cell differentiation, is basically maintained in memory Th2 cells (Figure 2). Memory Th2 cells are reported to maintain their Th2 signatures, specifically the Th2 cytokine production ability upon recall TCR stimulation and permissive histone modifications at the Th2 cytokine gene loci. These signatures are maintained by the high-level expression of GATA3 in an IL-4-independent manner (14, 59, 73–75). In addition, the expression of Th2 cytokine genes in memory Th2 cells depends on GATA3, since *Gata3* knockdown diminishes the transcription of these and other Th2-specific genes (52, 58). When TrxG proteins are genetically depleted, memory Th2 cells fail to maintain the *Gata3* expression and produce reduced amounts of Th2 cytokines after TCR stimulation due to the decreased methylation of H3K4 and the acetylation of H3K9. For example, the decreased expression of *Gata3* and impaired type 2 immune responses are observed in *Kmt2a*^+/−^ (referred to as MLL1^+/−^ elsewhere in this review) mice (76). Menin-deficient memory Th2 cells show a similar but milder phenotype (25). This is probably due to the redundancy of Menin, which is reported to only be included in the MLL1/2-bearing TrxG complex. In addition to the *Gata3* gene locus, permissive histone marks in Th2 cytokine gene loci are proposed to be maintained by MLL1 and Menin. In fact, the direct binding of MLL1 and Menin is detected at specific regions of Th2 cytokine gene loci as well as at the *Gata3* gene locus. Th2 cytokine production is dramatically reduced in MLL1^+/−^ memory Th2 cells in concurrence with decreased levels of the permissive histone marks, including H3K9 acetylation and H3K4 methylation. Accordingly, MLL1^+/−^ memory Th2 cells have a compromised ability to induce antigen-dependent allergic airway inflammation *in vivo* in comparison to wild-type control cells, suggesting a pathophysiological role of MLL1 in allergic diseases. Thus, TrxG molecules MLL1 and Menin epigenetically stabilize and maintain the *Gata3* mRNA expression in memory Th2 cells (14).

### The PcG Protein Bmi1 Regulates the Survival of Memory Th2 Cells

As described above, it is generally thought that some of the effector Th cells that survive after antigen clearance are a major source of memory Th cells. Thus, the mechanism underlying the survival of memory Th2 cells is an important issue to be addressed. It has been proposed that the PcG protein Bmi1 (also called Pcgf4) is responsible for the survival of memory Th2 cells as well as the self-renewal of hematopoietic stem cells (77). Indeed, a Bmi1-dependent (Bmi1^+/+^, Bmi1^+/−^, and Bmi1^−/−^ were compared) decrease was observed in the numbers of memory Th2 cells. In hematopoietic stem cells, Bmi1 exerts its function via the repression of Ink4a/Arf, which are produced by different isoforms of the *Cdkn2a* gene (78, 79). However, the Bmi1-dependent repression of Noxa, which is encoded by the *Pmaip1* gene, is required to prevent apoptosis in memory Th2 cells (77, 80). Bmi1 binds to the CpG islands of the *Pmaip1* gene along with other PcG proteins (Ring1B and Suz12) and suppresses the gene expression via H3K27 methylation. In addition, Bmi1 recruits DNA methyl transferase 1 (Dnmt1) to preserve CpG methylation of the *Pmaip1* gene (77, 81). Thus, Bmi1 modulates the memory Th2 cell survival through the repression of the *Pmaip1* gene.

## The Heterogeneity of Memory Th2 Cells

### The Identification of Pathogenic Th2 (Tpath2) Cells With Distinctive Epigenetic Modifications

Although we have described molecular mechanisms underlying the maintenance of the memory Th2 cell functions based on the analysis of the “bulk” cell population, recent advances in experimental techniques have enabled us to analyze the expression of proteins and transcripts at the “single cell” level *in vivo* (82). These analyses revealed that the cell populations (e.g., hematopoietic stem cell) in our body are much more heterogeneous than initially believed (83). The abovementioned memory Th2 cells also show heterogeneity and can be classified into subpopulations by the expression patterns of cell surface molecules, such as chemokine receptors and cell adhesion molecules. Among these subpopulations, we discovered that one population in which memory Th2 cells express low levels of both chemokine receptor CXCR3 and cell adhesion molecule CD62L (CD62L^lo^CXCR3^lo^) produces a large amount of IL-5, which is closely related to the pathogenesis of eosinophilic airway inflammation (84). The IL-5 secretion from CD62L^lo^CXCR3^lo^ memory Th2 cells is strictly regulated by histone modifications and the expression of the transcription factor Eomes. In this population, permissive histone modifications, including H3K4 trimethylation are observed at the promotor region of the *Il5* gene locus. Furthermore, the Eomes expression of CD62L^lo^CXCR3^lo^ memory Th2 cells is very low, which inhibits the binding of GATA3 to the *Il5* promotor and the subsequent *Il5* transcriptional induction in other populations. Thus, these cells are capable of producing a large amount of IL-5 in response to antigenic stimulation. CD62L^lo^CXCR3^lo^ memory Th2 cells, which produce large amounts of IL-5, recruit eosinophils to inflammatory tissues *in vivo* and are closely related to the pathogenicity of eosinophilic airway inflammation. Thus, we named these pathogenic memory Th2 (memory Tpath2) cells (84, 85). Another group reported that chemokine receptor CCR8-positive Th2 cells can produce large amounts of IL-5 and are involved in the pathogenicity of chronic atopic dermatitis in a mouse model (86). These reports raise the possibility that memory Tpath2 cells can be further classified into subpopulations with distinctive chromatin modifications that might be related to the pathogenicity of each disease.

### The Induction and Maintenance Mechanisms of Tpath2 Cells

**Figure 3:**
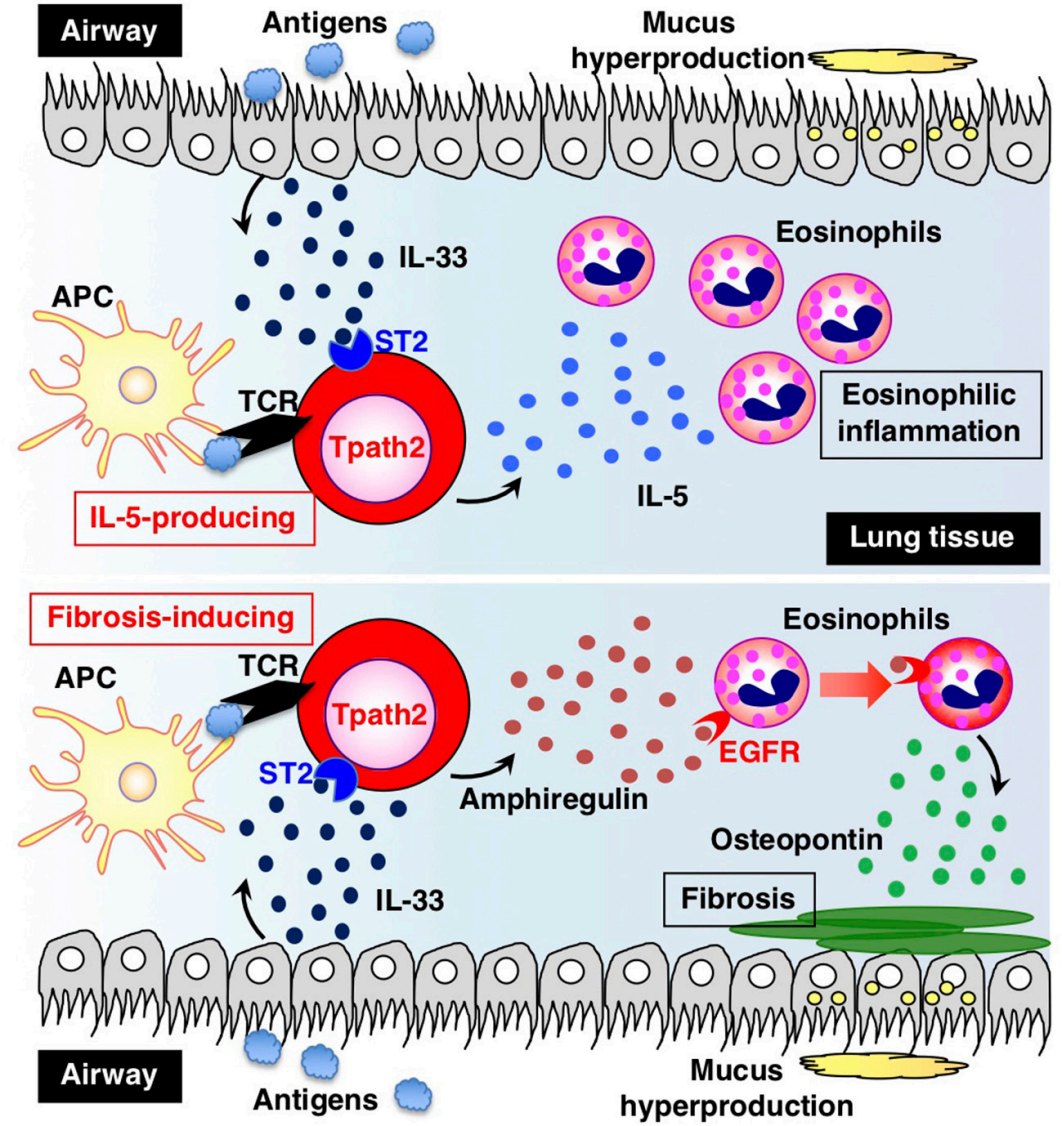
The two types of pathogenic memory Th2 (Tpath2) cells. Antigens that are potentially associated with allergic reactions promote the secretion of IL-33 from airway epithelial cells. IL-33 binds to its receptor ST2 on memory Th2 cells and induces epigenetic changes of the *Il5* gene, resulting in the generation of IL-5-producing Tpath2 cells (upper) (1, 87). When Tpath2 cells reencounter their cognate antigens, these cells produce a large amount of IL-5, which exacerbates chronic eosinophilic inflammation in the lung. IL-33 also induces the production of fibrosis-inducing Tpath2 cells, which produce large amounts of Amphiregulin (lower) (88). Amphiregulin binds to the epidermal growth factor receptors (EGFRs) on eosinophils and induces Osteopontin secretion from the eosinophils, resulting in fibrosis in the lung tissue. This figure was reproduced according to the permissions policy of Cell Press journal [originally published by Morimoto et al. (88)].

How are Tpath2 cells that produce large amounts of IL-5 induced *in vivo*? It is proposed that epithelial cytokines, including IL-25, IL-33, and thymic stromal lymphopoietin (TSLP), which are released from the epithelial cells of the respiratory tract, play an important role in inducing Tpath2 cell differentiation (Figure 3). These cytokines have an “alarmin” function and induce an inflammatory response in the mucosal membrane. Our study revealed that *in vivo*, memory Th2 cells express elevated levels of IL-33 receptor ST2 compared to differentiated effector Th2 cells *in vitro* (87). Indeed, IL-33 stimulation activates memory Th2 cells and induces a large amount of IL-5 production via chromatin remodeling at the *Il5* gene locus. Interestingly, IL-33 stimulation also induces chromatin remodeling at the *Il1rl1* gene locus, which encodes ST2, resulting in the increased expression of ST2 in memory Th2 cells. An RNA-seq analysis of gene expression patterns induced by IL-33 stimulation in memory Tpath2 cells identified other candidate molecules responsible for eosinophilic inflammation. Amphiregulin, which is encoded by the *Areg* gene, has been reported to be associated with tissue repair and fibrosis and was one of the candidates identified by this analysis (88). Fibrosis around the airway, which is often found in patients with chronic airway inflammation, is typically formed in an airway inflammation mouse model induced by house dust mite (HDM). Thus, we hypothesized that the IL-33-Amphreglin axis has a pathogenic function to induce fibrosis in airway inflammation. Indeed, IL-33 stimulation induced permissive histone modifications at the *Areg* gene locus *in vitro*. The deletion of the *Areg* gene resulted in the attenuation of the lung fibrosis induced by Tpath2 cells. Amphiregulin had a direct effect on epidermal growth factor receptors (EGFRs) on eosinophils, which causes them to produce Osteopontin, which induces fibrosis. Thus, a subpopulation of Tpath2 cells that produce Amphiregulin functions as “fibrosis inducing memory Tpath2 cells” (Figure 3). Although Tpath2 cells and ILC2 cells share some signatures, including the ability to produce IL-5, they differ in responsiveness to IL-33 stimulation. ILC2 cells can produce IL-5 in response to IL-33 stimulation whereas Tpath2 cells need TCR stimulation to produce IL-5. Dusp10, which is highly expressed in Tpath2 cells compared to ILC2 cells, was found to be involved in inhibiting IL-33-dependent IL-5 production in Tpath2 cells (89). Thus, the Dusp10-mediated suppression of IL-5 may explain the difference in responsiveness to IL-33 between Tpath2 and ILC2. Most recently, CXCR6^+^ST2^+^ memory Th2 cells have been found to exert a protective function in immunity against helminth infection (90). This finding supports the hygiene hypothesis that lack of exposure to parasites increases susceptibility to allergic diseases: ST2^+^ memory Th2 cells play a protective role against helminth infection but play a pathogenic role in allergic reactions in the absence of parasite infection.

In addition to IL-33, IL-7 also plays a role in the maintenance of memory Th2 cell functions in an ectopic lymphoid tissue called “inducible bronchus-associated lymphoid tissue” (iBALT) (91). The chronic inflammation caused by various factors such as infectious diseases, smoking, and collagen diseases is reported to induce the formation of iBALT in the lung (91). Notably, Thy1 (a cell surface molecule)-positive lymphatic endothelial cells produce IL-7 in the inflamed lung tissue and are essential for the formation of iBALT and memory Tpath2 cell maintenance in iBALT. More interestingly, Thy1-positive IL-7-producing lymphatic endothelial cells in iBALT also strongly express IL-33 and are implicated in the maintenance of the memory Tpath2 cell function in iBALT (91). Taken together, these findings suggest that memory Tpath2 cells develop from memory Th2 cells *in vivo* via epigenetic mechanisms in the presence of an environmental signal molecule (IL-33) and are maintained by receiving signals that are important for their functional maintenance and survival in the inflamed tissues microenvironment of iBALT, which is proposed to be an “inflammation niche.”

## The Regulation of the Recall-responses of Effector and Memory Th2 Cells

### The Acute Immune Response in the Airway Mediated by Effector Th2 Cells Is Dependent on CD69 and its Ligand Myl9/12

**Figure 4:**
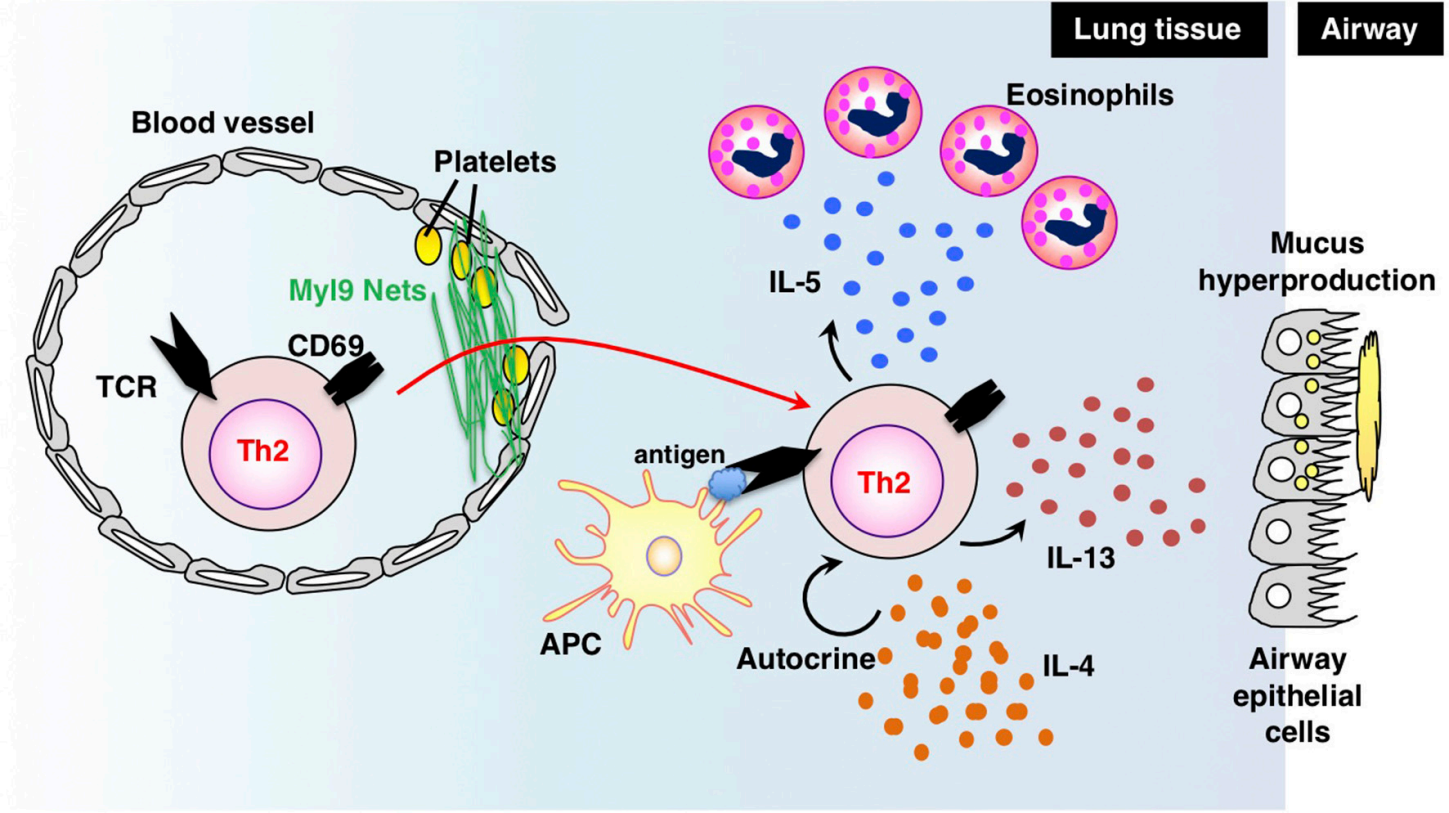
The “CD69-Myl9 system.” In the inflamed lung, platelet-derived Myl9 forms a net-like structure in association with platelets on the luminal surface of blood vessels (92, 93). CD69 molecules on effector Th2 cells interact with Myl9 nets and help Th2 cells infiltrate tissues. Infiltrating Th2 cells reencounter their cognate antigens and exert effector functions through the production of Th2 cytokines. IL-5 is known to recruit eosinophils whereas IL-13 promotes mucus hyperproduction from airway epithelial cells. IL-4 stimulates Th2 proliferation in an autocrine manner. This figure was reproduced according to the permissions policy of John Wiley & Sons publications [originally published by Kimura et al. (93)].

Antigen-primed Th cells migrate to inflamed sites via the blood stream and infiltrate inflammatory tissues through vessels. Thus, migration into inflammatory tissues, where Th cells reencounter their cognate antigens, is important for Th cells to exert their effector functions in acute immune responses. In a recent study, we successfully identified myosin light chain (Myl9/12) as a functional ligand for CD69 and proposed a new migration mechanism that is dependent on interaction between CD69 and Myl9 (the “CD69-Myl9 system”) (92, 93) (Figure 4). CD69 was originally identified as a molecule that is rapidly induced on T, B, and NK cells upon activation (93). CD69 is a type 2 cell membrane protein with a C-type lectin-like domain. TCR stimulation increases H3K4 methylation at the *Cd69* gene in naïve CD4 T cells, suggesting that the expression of CD69 is epigenetically regulated (94). More recently, CD69 has been found to be crucial for maturation of NKT2 cells in the thymus, where CD69 prevents immature precursors from exiting by suppressing the sphingosine-1-phosphate receptor 1 (S1P_1_) expression (95). A number of studies have reported roles of CD69 in murine models of inflammatory diseases, including arthritis, airway inflammation, and dextran sulfate sodium (DSS)-induced colitis (96–98). However, the CD69 ligand had not been identified before our report on Myl9/12. We found that Myl9/12 molecules are released from platelets in inflammatory vessels and then form net-like structures (Myl9 nets) that help activated immune cells infiltrate the blood vessels and migrate into inflammatory tissues. Myl9/12 monoclonal antibody (Ab) treatment was proven to be effective in both OVA-induced and HDM-induced airway inflammation models. These results suggest that anti-Myl9/12 Abs-based antibody therapy may also be useful for severe steroid-resistant asthma treatment in humans, and humanized anti-Myl9/12 Abs that can be administered to humans are now being prepared.

### The Epigenetic Regulation of the Recall-Responses of Memory Th2 Cells by the TrxG Proteins MLL1 and Menin

As described in a previous section, TrxG proteins, such as MLL1 and Menin maintain H3K4 methylation of the *Gata3* and Th2 cytokine genes and are crucial for the rapid recall response of memory Th2 cells. Menin is also indispensable for the survival of memory Th2 cells because ablation of Menin is shown to significantly decrease the number of memory Th2 cells. However, even in an experimental setting where the same number of wild-type and Menin-deficient memory Th2 cells are transferred into congenic mice, which are challenged by OVA, the deletion of Menin attenuates airway inflammation, indicating that the Menin-dependent regulation of the Th2 signature genes is important for type 2 immune responses (25). TrxG proteins are involved in both pathogenic and protective immune responses. For example, MLL1 is reported to play a role in the anti-tumor immunity mediated by memory Th2 cells (99). Thus, TrxG proteins are required for both keeping the epigenetic states active in the Th2 signature genes and for preventing programmed cell death of memory Th2 cells, both of which are essential for a proper recall response to antigens.

Menin is also needed for the long-term maintenance of the Th2 cell identity and a proper response to antigen restimulation when Th2 cells are exposed to antigen multiple times *in vitro*. Th2 cells subjected to TCR stimulation multiple times are reported to produce higher levels of IL-5 and IL-13 *in vitro* than normal effector Th2 cells (25). In contrast, IL-4 production is slightly increased by multiple TCR stimulation, which is required for the complete demethylation of CpGs of the *Il4* gene (100). In these established Th2 cells, the deletion of Menin decreased the expression of Th2 signature genes, including the *Gata3* and Th2 cytokine genes (25). *In vivo*, multiple exposure to an antigen has a different effect: the pathophysiology of airway inflammation changes from Th2-mediated to Th1- and Th17-mediated inflammation. Th17-mediated airway inflammation is known to be associated with steroid-resistant asthma (101). Menin has been implicated in the pathogenesis of airway inflammation in a mouse model resembling steroid-resistant asthma (102). Menin also plays a role in the protective immune response to listeria infection in CD8 T cells (103). Thus, epigenetic regulation mediated by TrxG proteins is important for both pathogenic and protective immune responses.

### The Involvement of Tpath2 Cells in Human Chronic Allergic Diseases

In previous sections, we focused on a mouse model of airway inflammation associated with Th2-mediated inflammatory diseases. In this section, we discuss the recent findings concerning human chronic allergic diseases, with a focus on chronic rhinosinusitis (CRS), which is one of the most common complications of bronchial asthma (1). CRS refers to a type of chronic upper respiratory tract inflammation that is characterized by the inflammation of the mucosa of the nasal and paranasal cavity and tissue remodeling. The pathogenesis of CRS and the process through which the inflammation of CRS develops are thought to be similar to those of bronchial asthma, which is caused by lower respiratory tract inflammation. CRS is categorized into two groups according to the presence or absence of nasal polyps (NPs): CRS without NPs (CRSsNPs) and CRS with NPs (CRSwNPs) (1). Polyps from CRSwNP patients usually contain large numbers of infiltrating eosinophils and are thought to be a local lesion of chronic eosinophilic inflammation. Thus, CRSwNPs is also called eosinophilic CRS (ECRS). The pathophysiology of ECRS is unclear at present; we analyzed polyp-infiltrating T cells and found that the polyps of eosinophilic rhinosinusitis patients contain large numbers of infiltrating memory CD4 T cells that secrete large amounts of IL-5 in response to IL-33 stimulation (87, 91). These memory CD4 T cells strongly express IL-17 receptor B (IL-17RB), which is a receptor of IL-25 and involved in IL-5 production in response to IL-25 stimulation (104). In addition, CD69-expressing T cells and Myl9 nets have been identified within the polyps, indicating that the “CD69-Myl9 system” plays a role in the pathogenesis of ECRS (92, 93). Consistent with the mouse model of airway inflammation, ectopic lymphoid tissues with Thy1-positive IL-7-producing lymphatic endothelial cells are formed in ECRS polyps (91). In addition, fibrosis is also observed in ECRS polyps. A further analysis revealed that memory Th2 cells that highly express the cell surface molecules CD161 and CRTH2 specifically produce IL-5 and Amphiregulin *in vivo* (88). Other research groups have reported that Tpath2 cells also contribute to allergic reactions in the gastrointestinal tract, such as human eosinophilic esophagitis and food allergy (105). In addition, it is reported that sublingual immunotherapy can reduce the number of Tpath2 cells in the peripheral blood of pollinosis patients (106). These results suggest that the IL-33-dependent induction of memory Tpath2 cells is closely associated with chronic inflammation in both humans and mice.

## Concluding Remarks and Open Questions to be Addressed

Extensive research on Th2 cells has shed light on the epigenetic regulation in the induction, maintenance, heterogeneity, and recall-response of memory T cells. For the induction of Th2 cells, STAT6 regulates epigenetic changes of the *Gata3* gene, resulting in the expression of extremely high levels of GATA3 proteins, which control chromatin remodeling at Th2 cytokine gene loci. STAT6 and GATA3 also recruit TrxG H3K4 methylase proteins to the appropriate regions of the *Gata3* and Th2 cytokine gene loci, respectively. The recruited TrxG proteins are required for the maintenance of the high expression of the *Gata3* gene and the production of Th2 cytokines in memory Th2 cells upon secondary TCR stimulation, indicating that the recall-response of memory Th2 cells is also dependent on epigenetic machinery. Memory Th2 cells show heterogeneity and can be classified into subpopulations with distinctive epigenetic modifications. For example, CD62L^lo^CXCR3^lo^ Tpath2 cells produce a large amount of IL-5, whereas a subpopulation of the Tpath2 cells produces Amphiregulin and is involved in fibrosis in the airway of mice and humans. Taken together, these findings suggest that allergic airway inflammation is caused by a certain subpopulation of memory Th2 cells or a combination of subpopulations. The “pathogenic Th population disease induction model” we have proposed may thus explain the pathogenesis of allergic airway inflammation more accurately than the classical model, in which an imbalance in Th1/Th2 differentiation is proposed to be responsible for allergic disease (1, 85).

Various important and interesting questions remain to be addressed. The first question is how a small number of memory T cells are selected from a large number of effector T cells. Some reports show that effector T cells harboring TCRs with a low affinity to antigens are prone to survive and form a memory T cell population, while other reports argue that some naïve T cells are directly differentiated into memory precursor cells after antigen priming (107, 108). Another question is where memory T cells are located. Previously inflamed tissue, draining lymph nodes, other secondary lymphoid organs, the bone marrow, and the peripheral blood are potential locations (109). Regarding epigenetics, histone modifications and DNA methylation states are reported to be preserved from effector T cells to memory T cells (110). However, the extent to which the three-dimensional structures of the epigenome are maintained in memory T cells in comparison to effector T cells is not clear. For example, whether chromatin structures, interactions between enhancers and promoters and genomic locations in the nucleus are maintained, resolved, or renewed remains to be determined. Future mechanistic studies, including kinetic analyses of cell migration and cell-intrinsic changes will be needed to improve our understanding of memory T cell biology and epigenomics.

## Author Contributions

All authors listed have made a substantial, direct and intellectual contribution to the work, and approved it for publication.

### Conflict of Interest Statement

The authors declare that the research was conducted in the absence of any commercial or financial relationships that could be construed as a potential conflict of interest.

## References

[1] Nakayama T Hirahara K Onodera A Endo Y Hosokawa H Shinoda K. Th2 Cells in Health and Disease. Annu Rev Immunol. (2017) 35:53–84. 10.1146/annurev-immunol-051116-052350 27912316

[2] Onodera A Tumes DJ Nakayama T Epigenetic control of immune T cell memory. In: Bonifer C, Cockerill PN, editors. Transcriptional and Epigenetic Mechanisms Regulating Normal and Aberrant Blood Cell Development. Epigenetics and Human Health. (Berlin, Heidelberg: Springer). p. 367–82.

[3] Chang JT Wherry EJ Goldrath AW. Molecular regulation of effector and memory T cell differentiation. Nat Immunol. (2014) 15:1104–15. 10.1038/ni.3031 25396352 PMC4386685

[4] Kaech SM Wherry EJ Ahmed R. Effector and memory T-cell differentiation: implications for vaccine development. Nat Rev Immunol. (2002) 2:251–62. 10.1038/nri778 12001996

[5] Bevington SL Cauchy P Withers DR Lane PJL Cockerill PN. T cell receptor and cytokine signaling can function at different stages to establish and maintain transcriptional memory and enable T helper cell differentiation. Front Immunol. (2017) 8:204. 10.3389/fimmu.2017.00204 28316598 PMC5334638

[6] Zhu J Yamane H Paul WE. Differentiation of effector CD4 T cell populations (^*^). Annu Rev Immunol. (2010) 28:445–89. 10.1146/annurev-immunol-030409-101212 20192806 PMC3502616

[7] Oliveira AL de B Monteiro VVS Navegantes-Lima KC Reis JF Gomes RS Rodrigues DVS. Resveratrol role in autoimmune disease-a mini-review. Nutrients (2017) 9:E1306. 10.3390/nu9121306 29194364 PMC5748756

[8] Stockinger B Omenetti S. The dichotomous nature of T helper 17 cells. Nat Rev Immunol. (2017) 17:535–44. 10.1038/nri.2017.50 28555673

[9] Wilson CB Rowell E Sekimata M. Epigenetic control of T-helper-cell differentiation. Nat Rev Immunol. (2009) 9:91–105. 10.1038/nri2487 19151746

[10] Turner BM. Cellular memory and the histone code. Cell (2002) 111:285–91. 10.1016/S0092-8674(02)01080-2 12419240

[11] Smith ZD Meissner A. DNA methylation: roles in mammalian development. Nat Rev Genet. (2013) 14:204–20. 10.1038/nrg3354 23400093

[12] Peschansky VJ Wahlestedt C. Non-coding RNAs as direct and indirect modulators of epigenetic regulation. Epigenetics (2014) 9:3–12. 10.4161/epi.27473 24739571 PMC3928183

[13] Onodera A Nakayama T. Epigenetics of T cells regulated by Polycomb/Trithorax molecules. Trends Mol Med. (2015) 21:330–40. 10.1016/j.molmed.2015.03.001 25842254

[14] Nakayama T Yamashita M. Critical role of the Polycomb and Trithorax complexes in the maintenance of CD4 T cell memory. Semin Immunol. (2009) 21:78–83. 10.1016/j.smim.2009.02.001 19269851

[15] Schuettengruber B Bourbon H-M Di Croce L Cavalli G. Genome Regulation by Polycomb and Trithorax: 70 years and counting. Cell (2017) 171:34–57. 10.1016/j.cell.2017.08.002 28938122

[16] Mohan M Herz H-M Shilatifard A. SnapShot: histone lysine methylase complexes. Cell (2012) 149:498.e1. 10.1016/j.cell.2012.03.025 22500810 PMC3711870

[17] Hu D Garruss AS Gao X Morgan MA Cook M Smith ER. The Mll2 branch of the COMPASS family regulates bivalent promoters in mouse embryonic stem cells. Nat Struct Mol Biol. (2013) 20:1093–7. 10.1038/nsmb.2653 23934151 PMC3805109

[18] Schuettengruber B Martinez A-M Iovino N Cavalli G. Trithorax group proteins: switching genes on and keeping them active. Nat Rev Mol Cell Biol. (2011) 12:799–814. 10.1038/nrm3230 22108599

[19] Takeda K Tanaka T Shi W Matsumoto M Minami M Kashiwamura S. Essential role of Stat6 in IL-4 signalling. Nature (1996) 380:627–30. 10.1038/380627a0 8602263

[20] Kaplan MH Schindler U Smiley ST Grusby MJ. Stat6 is required for mediating responses to IL-4 and for development of Th2 cells. Immunity (1996) 4:313–9. 10.1016/S1074-7613(00)80439-2 8624821

[21] Wei L Vahedi G Sun H-W Watford WT Takatori H Ramos HL. Discrete roles of STAT4 and STAT6 transcription factors in tuning epigenetic modifications and transcription during T helper cell differentiation. Immunity (2010) 32:840–51. 10.1016/j.immuni.2010.06.003 20620946 PMC2904651

[22] Onodera A Yamashita M Endo Y Kuwahara M Tofukuji S Hosokawa H. STAT6-mediated displacement of polycomb by trithorax complex establishes long-term maintenance of GATA3 expression in T helper type 2 cells. J Exp Med. (2010) 207:2493–506. 10.1084/jem.20100760 20956546 PMC2964576

[23] Onodera A Tumes DJ Watanabe Y Hirahara K Kaneda A Sugiyama F. Spatial interplay between Polycomb and Trithorax complexes controls transcriptional activity in T lymphocytes. Mol Cell Biol. (2015) 35:3841–53. 10.1128/MCB.00677-15 26324324 PMC4609743

[24] Scheinman EJ Avni O. Transcriptional regulation of GATA3 in T helper cells by the integrated activities of transcription factors downstream of the interleukin-4 receptor and T cell receptor. J Biol Chem. (2009) 284:3037–48. 10.1074/jbc.M807302200 19056736

[25] Onodera A Kiuchi M Kokubo K Kato M Ogino T Horiuchi S. Menin controls the memory Th2 cell function by maintaining the epigenetic integrity of Th2 cells. J Immunol. (2017) 199:1153–62. 10.4049/jimmunol.1602129 28659357

[26] Wei G Wei L Zhu J Zang C Hu-Li J Yao Z. Global mapping of H3K4me3 and H3K27me3 reveals specificity and plasticity in lineage fate determination of differentiating CD4+ T cells. Immunity (2009) 30:155–67. 10.1016/j.immuni.2008.12.009 19144320 PMC2722509

[27] Bernstein BE Mikkelsen TS Xie X Kamal M Huebert DJ Cuff J. A bivalent chromatin structure marks key developmental genes in embryonic stem cells. Cell (2006) 125:315–26. 10.1016/j.cell.2006.02.041 16630819

[28] Tumes DJ Onodera A Suzuki A Shinoda K Endo Y Iwamura C. The polycomb protein Ezh2 regulates differentiation and plasticity of CD4(+) T helper type 1 and type 2 cells. Immunity (2013) 39:819–32. 10.1016/j.immuni.2013.09.012 24238339

[29] Mosmann TR Coffman RL. TH1 and TH2 cells: different patterns of lymphokine secretion lead to different functional properties. Annu Rev Immunol. (1989) 7:145–73. 10.1146/annurev.iy.07.040189.001045 2523712

[30] Reiner SL Locksley RM. The regulation of immunity to Leishmania major. Annu Rev Immunol. (1995) 13:151–77. 10.1146/annurev.iy.13.040195.001055 7612219

[31] Ho I-C Tai T-S Pai S-Y. GATA3 and the T-cell lineage: essential functions before and after T-helper-2-cell differentiation. Nat Rev Immunol. (2009) 9:125–35. 10.1038/nri2476 19151747 PMC2998182

[32] Yagi R Zhong C Northrup DL Yu F Bouladoux N Spencer S. The transcription factor GATA3 is critical for the development of all IL-7Rα-expressing innate lymphoid cells. Immunity (2014) 40:378–88. 10.1016/j.immuni.2014.01.012 24631153 PMC4026797

[33] Pai S-Y Truitt ML Ting C-N Leiden JM Glimcher LH Ho I-C. Critical roles for transcription factor GATA-3 in thymocyte development. Immunity (2003) 19:863–75. 10.1016/S1074-7613(03)00328-5 14670303

[34] Hernández-Hoyos G Anderson MK Wang C Rothenberg EV Alberola-Ila J. GATA-3 expression is controlled by TCR signals and regulates CD4/CD8 differentiation. Immunity (2003) 19:83–94. 10.1016/S1074-7613(03)00176-6 12871641

[35] Yamamoto M Ko LJ Leonard MW Beug H Orkin SH Engel JD. Activity and tissue-specific expression of the transcription factor NF-E1 multigene family. Genes Dev. (1990) 4:1650–62. 10.1101/gad.4.10.1650 2249770

[36] Hosoya T Maillard I Engel JD. From the cradle to the grave: activities of GATA-3 throughout T-cell development and differentiation. Immunol Rev. (2010) 238:110–25. 10.1111/j.1600-065X.2010.00954.x 20969588 PMC2965564

[37] Wei G Abraham BJ Yagi R Jothi R Cui K Sharma S. Genome-wide analyses of transcription factor GATA3-mediated gene regulation in distinct T cell types. Immunity (2011) 35:299–311. 10.1016/j.immuni.2011.08.007 21867929 PMC3169184

[38] Wang L Wildt KF Zhu J Zhang X Feigenbaum L Tessarollo L. Distinct functions for the transcription factors GATA-3 and ThPOK during intrathymic differentiation of CD4(+) T cells. Nat Immunol. (2008) 9:1122–30. 10.1038/ni.1647 18776904 PMC2805063

[39] Ouyang W Ranganath SH Weindel K Bhattacharya D Murphy TL Sha WC. Inhibition of Th1 development mediated by GATA-3 through an IL-4-independent mechanism. Immunity (1998) 9:745–55. 10.1016/S1074-7613(00)80671-8 9846495

[40] Ansel KM Djuretic I Tanasa B Rao A. Regulation of Th2 differentiation and Il4 locus accessibility. Annu Rev Immunol. (2006) 24:607–56. 10.1146/annurev.immunol.23.021704.115821 16551261

[41] Buenrostro JD Giresi PG Zaba LC Chang HY Greenleaf WJ. Transposition of native chromatin for fast and sensitive epigenomic profiling of open chromatin, DNA-binding proteins and nucleosome position. Nat Methods (2013) 10:1213–8. 10.1038/nmeth.2688 24097267 PMC3959825

[42] Zheng W Flavell RA. The transcription factor GATA-3 is necessary and sufficient for Th2 cytokine gene expression in CD4 T cells. Cell (1997) 89:587–96. 10.1016/S0092-8674(00)80240-8 9160750

[43] Zhang DH Cohn L Ray P Bottomly K Ray A. Transcription factor GATA-3 is differentially expressed in murine Th1 and Th2 cells and controls Th2-specific expression of the interleukin-5 gene. J Biol Chem. (1997) 272:21597–603. 10.1074/jbc.272.34.21597 9261181

[44] Ouyang W Löhning M Gao Z Assenmacher M Ranganath S Radbruch A. Stat6-independent GATA-3 autoactivation directs IL-4-independent Th2 development and commitment. Immunity (2000) 12:27–37. 10.1016/S1074-7613(00)80156-9 10661403

[45] Horiuchi S Onodera A Hosokawa H Watanabe Y Tanaka T Sugano S. Genome-wide analysis reveals unique regulation of transcription of Th2-specific genes by GATA3. J Immunol. (2011) 186:6378–89. 10.4049/jimmunol.1100179 21536806

[46] Shinnakasu R Yamashita M Kuwahara M Hosokawa H Hasegawa A Motohashi S. Gfi1-mediated stabilization of GATA3 protein is required for Th2 cell differentiation. J Biol Chem. (2008) 283:28216–25. 10.1074/jbc.M804174200 18701459 PMC2661392

[47] Yamashita M Shinnakasu R Asou H Kimura M Hasegawa A Hashimoto K. Ras-ERK MAPK cascade regulates GATA3 stability and Th2 differentiation through ubiquitin-proteasome pathway. J Biol Chem. (2005) 280:29409–19. 10.1074/jbc.M502333200 15975924

[48] Hosokawa H Kato M Tohyama H Tamaki Y Endo Y Kimura MY. Methylation of Gata3 protein at Arg-261 regulates transactivation of the Il5 gene in T helper 2 cells. J Biol Chem. (2015) 290:13095–103. 10.1074/jbc.M114.621524 25861992 PMC4505565

[49] Shinnakasu R Yamashita M Shinoda K Endo Y Hosokawa H Hasegawa A. Critical YxKxHxxxRP motif in the C-terminal region of GATA3 for its DNA binding and function. J Immunol. (2006) 177:5801–10. 10.4049/jimmunol.177.9.5801 17056504

[50] Hosokawa H Tanaka T Suzuki Y Iwamura C Ohkubo S Endoh K. Functionally distinct Gata3/Chd4 complexes coordinately establish T helper 2 (Th2) cell identity. Proc Natl Acad Sci USA. (2013) 110:4691–6. 10.1073/pnas.1220865110 23471993 PMC3606997

[51] Fang D Cui K Hu G Gurram RK Zhong C Oler AJ. Bcl11b, a novel GATA3-interacting protein, suppresses Th1 while limiting Th2 cell differentiation. J Exp Med. (2018) 215:1449–62. 10.1084/jem.20171127 29514917 PMC5940260

[52] Onodera A Kokubo K Nakayama T The interplay between transcription factors and epigenetic modifications in Th2 cells. In: Fumiaki Uchiumi editor. Gene Expression and Regulation in Mammalian Cells - Transcription From General Aspects (London) (2018) 10.5772/intechopen.73027

[53] Yagi R Zhu J Paul WE. An updated view on transcription factor GATA3-mediated regulation of Th1 and Th2 cell differentiation. Int Immunol. (2011) 23:415–20. 10.1093/intimm/dxr029 21632975 PMC3123974

[54] Takemoto N Kamogawa Y Jun Lee H Kurata H Arai KI O'Garra A. Cutting edge: chromatin remodeling at the IL-4/IL-13 intergenic regulatory region for Th2-specific cytokine gene cluster. J Immunol. (2000) 165:6687–91. 10.4049/jimmunol.165.12.6687 11120785

[55] Takemoto N Arai K Miyatake S. Cutting edge: the differential involvement of the N-finger of GATA-3 in chromatin remodeling and transactivation during Th2 development. J Immunol. (2002) 169:4103–7. 10.4049/jimmunol.169.8.4103 12370337

[56] Agarwal S Avni O Rao A. Cell-type-restricted binding of the transcription factor NFAT to a distal IL-4 enhancer *in vivo*. Immunity (2000) 12:643–52. 10.1016/S1074-7613(00)80215-0 10894164

[57] Yamashita M Ukai-Tadenuma M Kimura M Omori M Inami M Taniguchi M. Identification of a conserved GATA3 response element upstream proximal from the interleukin-13 gene locus. J Biol Chem. (2002) 277:42399–408. 10.1074/jbc.M205876200 12205084

[58] Sasaki T Onodera A Hosokawa H Watanabe Y Horiuchi S Yamashita J. Genome-wide gene expression profiling revealed a critical role for GATA3 in the maintenance of the Th2 cell identity. PLoS ONE (2013) 8:e66468. 10.1371/journal.pone.0066468 23824597 PMC3688927

[59] Nakayama T Yamashita M. Initiation and maintenance of Th2 cell identity. Curr Opin Immunol. (2008) 20:265–71. 10.1016/j.coi.2008.03.011 18502111

[60] Tanaka S Motomura Y Suzuki Y Yagi R Inoue H Miyatake S. The enhancer HS2 critically regulates GATA-3-mediated Il4 transcription in T(H)2 cells. Nat Immunol. (2011) 12:77–85. 10.1038/ni.1966 21131966

[61] Kishikawa H Sun J Choi A Miaw SC Ho IC. The cell type-specific expression of the murine IL-13 gene is regulated by GATA-3. J Immunol. (2001) 167:4414–20. 10.4049/jimmunol.167.8.4414 11591766

[62] Schwenger GT Fournier R Kok CC Mordvinov VA Yeoman D Sanderson CJ. GATA-3 has dual regulatory functions in human interleukin-5 transcription. J Biol Chem. (2001) 276:48502–9. 10.1074/jbc.M107836200 11579103

[63] Lee HJ O'Garra A Arai K Arai N. Characterization of cis-regulatory elements and nuclear factors conferring Th2-specific expression of the IL-5 gene: a role for a GATA-binding protein. J Immunol. (1998) 160:2343–52. 9498775

[64] Goll MG Bestor TH. Eukaryotic cytosine methyltransferases. Annu Rev Biochem. (2005) 74:481–514. 10.1146/annurev.biochem.74.010904.153721 15952895

[65] Lee PP Fitzpatrick DR Beard C Jessup HK Lehar S Makar KW. A critical role for Dnmt1 and DNA methylation in T cell development, function, and survival. Immunity (2001) 15:763–74. 10.1016/S1074-7613(01)00227-8 11728338

[66] Makar KW Wilson CB DNA methylation is a non-redundant repressor of the Th2 effector program. J Immunol. (2004) 173:4402–6. 10.4049/jimmunol.173.7.4402 15383570

[67] Makar KW Pérez-Melgosa M Shnyreva M Weaver WM Fitzpatrick DR Wilson CB. Active recruitment of DNA methyltransferases regulates interleukin 4 in thymocytes and T cells. Nat Immunol. (2003) 4:1183–90. 10.1038/ni1004 14595437

[68] Lee YJ Holzapfel KL Zhu J Jameson SC Hogquist KA. Steady-state production of IL-4 modulates immunity in mouse strains and is determined by lineage diversity of iNKT cells. Nat Immunol. (2013) 14:1146–54. 10.1038/ni.2731 24097110 PMC3824254

[69] Kwon D-I Lee YJ. Lineage differentiation program of invariant natural killer T cells. Immune Netw. (2017) 17:365–77. 10.4110/in.2017.17.6.365 29302250 PMC5746607

[70] Kim PJ Pai S-Y Brigl M Besra GS Gumperz J Ho I-C. GATA-3 regulates the development and function of invariant NKT cells. J Immunol. (2006) 177:6650–9. 10.4049/jimmunol.177.10.6650 17082577

[71] Yasuoka T Kuwahara M Yamada T Maruyama S Suzuki J Taniguchi M. The transcriptional repressor Gfi1 plays a critical role in the development of NKT1- and NKT2-Type iNKT cells. PLoS ONE (2016) 11:e0157395. 10.1371/journal.pone.0157395 27284976 PMC4902269

[72] Hale JS Ahmed R. Memory T follicular helper CD4 T cells. Front Immunol. (2015) 6:16. 10.3389/fimmu.2015.00016 25699040 PMC4313784

[73] Yamashita M Shinnakasu R Nigo Y Kimura M Hasegawa A Taniguchi M. Interleukin (IL)-4-independent maintenance of histone modification of the IL-4 gene loci in memory Th2 cells. J Biol Chem. (2004) 279:39454–64. 10.1074/jbc.M405989200 15258154

[74] Yamashita M Ukai-Tadenuma M Miyamoto T Sugaya K Hosokawa H Hasegawa A. Essential role of GATA3 for the maintenance of type 2 helper T (Th2) cytokine production and chromatin remodeling at the Th2 cytokine gene loci. J Biol Chem. (2004) 279:26983–90. 10.1074/jbc.M403688200 15087456

[75] Pai S-Y Truitt ML Ho I-C. GATA-3 deficiency abrogates the development and maintenance of T helper type 2 cells. Proc Natl Acad Sci USA. (2004) 101:1993–8. 10.1073/pnas.0308697100 14769923 PMC357040

[76] Yamashita M Hirahara K Shinnakasu R Hosokawa H Norikane S Kimura MY. Crucial role of MLL for the maintenance of memory T helper type 2 cell responses. Immunity (2006) 24:611–22. 10.1016/j.immuni.2006.03.017 16713978

[77] Yamashita M Kuwahara M Suzuki A Hirahara K Shinnaksu R Hosokawa H. Bmi1 regulates memory CD4 T cell survival via repression of the Noxa gene. J Exp Med. (2008) 205:1109–20. 10.1084/jem.20072000 18411339 PMC2373843

[78] Oguro H Iwama A Morita Y Kamijo T van Lohuizen M Nakauchi H. Differential impact of Ink4a and Arf on hematopoietic stem cells and their bone marrow microenvironment in Bmi1-deficient mice. J Exp Med. (2006) 203:2247–53. 10.1084/jem.20052477 16954369 PMC2118102

[79] Iwama A Oguro H Negishi M Kato Y Morita Y Tsukui H. Enhanced self-renewal of hematopoietic stem cells mediated by the polycomb gene product Bmi-1. Immunity (2004) 21:843–51. 10.1016/j.immuni.2004.11.004 15589172

[80] Abdouh M Chatoo W El Hajjar J David J Ferreira J Bernier G. Bmi1 is down-regulated in the aging brain and displays antioxidant and protective activities in neurons. PLoS ONE (2012) 7:e31870. 10.1371/journal.pone.0031870 22384090 PMC3285640

[81] Negishi M Saraya A Miyagi S Nagao K Inagaki Y Nishikawa M. Bmi1 cooperates with Dnmt1-associated protein 1 in gene silencing. Biochem Biophys Res Commun. (2007) 353:992–8. 10.1016/j.bbrc.2006.12.166 17214966

[82] Kunz DJ Gomes T James KR. Immune cell dynamics unfolded by single-cell technologies. Front Immunol. (2018) 9:1435. 10.3389/fimmu.2018.01435 29997618 PMC6028612

[83] Wilson NK Kent DG Buettner F Shehata M Macaulay IC Calero-Nieto FJ. Combined single-cell functional and gene expression analysis resolves heterogeneity within stem cell populations. Cell Stem Cell (2015) 16:712–24. 10.1016/j.stem.2015.04.004 26004780 PMC4460190

[84] Endo Y Iwamura C Kuwahara M Suzuki A Sugaya K Tumes DJ. Eomesodermin controls interleukin-5 production in memory T helper 2 cells through inhibition of activity of the transcription factor GATA3. Immunity (2011) 35:733–45. 10.1016/j.immuni.2011.08.017 22118525

[85] Endo Y Hirahara K Yagi R Tumes DJ Nakayama T. Pathogenic memory type Th2 cells in allergic inflammation. Trends Immunol. (2014) 35:69–78. 10.1016/j.it.2013.11.003 24332592

[86] Islam SA Chang DS Colvin RA Byrne MH McCully ML Moser B. Mouse CCL8, a CCR8 agonist, promotes atopic dermatitis by recruiting IL-5+ T(H)2 cells. Nat Immunol. (2011) 12:167–77. 10.1038/ni.1984 21217759 PMC3863381

[87] Endo Y Hirahara K Iinuma T Shinoda K Tumes DJ Asou HK. The interleukin-33-p38 kinase axis confers memory T helper 2 cell pathogenicity in the airway. Immunity (2015) 42:294–308. 10.1016/j.immuni.2015.01.016 25692703

[88] Morimoto Y Hirahara K Kiuchi M Wada T Ichikawa T Kanno T. Amphiregulin-producing pathogenic memory T helper 2 cells instruct eosinophils to secrete osteopontin and facilitate airway fibrosis. Immunity (2018) 49:134–50.e6. 10.1016/j.immuni.2018.04.023 29958800

[89] Yamamoto T Endo Y Onodera A Hirahara K Asou HK Nakajima T. DUSP10 constrains innate IL-33-mediated cytokine production in ST2hi memory-type pathogenic Th2 cells. Nat Commun. (2018) 9:4231. 10.1038/s41467-018-06468-8 30315197 PMC6185962

[90] Obata-Ninomiya K Ishiwata K Nakano H Endo Y Ichikawa T Onodera A. CXCR6+ST2+ memory T helper 2 cells induced the expression of major basic protein in eosinophils to reduce the fecundity of helminth. Proc Natl Acad Sci USA. (2018) 115:E9849–58. 10.1073/pnas.1714731115 30275296 PMC6196506

[91] Shinoda K Hirahara K Iinuma T Ichikawa T Suzuki AS Sugaya K. Thy1+IL-7+ lymphatic endothelial cells in iBALT provide a survival niche for memory T-helper cells in allergic airway inflammation. Proc Natl Acad Sci USA. (2016) 113:E2842–51. 10.1073/pnas.1512600113 27140620 PMC4878506

[92] Hayashizaki K Kimura MY Tokoyoda K Hosokawa H Shinoda K Hirahara K. Myosin light chains 9 and 12 are functional ligands for CD69 that regulate airway inflammation. Sci Immunol. (2016) 1:eaaf9154. 10.1126/sciimmunol.aaf9154 28783682

[93] Kimura MY Hayashizaki K Tokoyoda K Takamura S Motohashi S Nakayama T. Crucial role for CD69 in allergic inflammatory responses: CD69-Myl9 system in the pathogenesis of airway inflammation. Immunol Rev. (2017) 278:87–100. 10.1111/imr.12559 28658550

[94] Allison KA Sajti E Collier JG Gosselin D Troutman TD Stone EL. Affinity and dose of TCR engagement yield proportional enhancer and gene activity in CD4+ T cells. eLife (2016) 5:e10134. 10.7554/eLife.10134 27376549 PMC4931909

[95] Kimura MY Igi A Hayashizaki K Mita Y Shinzawa M Kadakia T. CD69 prevents PLZFhi innate precursors from prematurely exiting the thymus and aborting NKT2 cell differentiation. Nat Commun. (2018) 9:3749. 10.1038/s41467-018-06283-1 30218105 PMC6138739

[96] Murata K Inami M Hasegawa A Kubo S Kimura M Yamashita M. CD69-null mice protected from arthritis induced with anti-type II collagen antibodies. Int Immunol. (2003) 15:987–92. 10.1093/intimm/dxg102 12882836

[97] Miki-Hosokawa T Hasegawa A Iwamura C Shinoda K Tofukuji S Watanabe Y. CD69 controls the pathogenesis of allergic airway inflammation. J Immunol. (2009) 183:8203–15. 10.4049/jimmunol.0900646 19923457

[98] Hasegawa A Iwamura C Kitajima M Hashimoto K Otsuyama K-I Ogino H. Crucial role for CD69 in the pathogenesis of dextran sulphate sodium-induced colitis. PLoS ONE (2013) 8:e65494. 10.1371/journal.pone.0065494 23785429 PMC3681816

[99] Kitajima M Ito T Tumes DJ Endo Y Onodera A Hashimoto K. Memory type 2 helper T cells induce long-lasting antitumor immunity by activating natural killer cells. Cancer Res. (2011) 71:4790–8. 10.1158/0008-5472.CAN-10-1572 21646476

[100] Lee DU Agarwal S Rao A. Th2 lineage commitment and efficient IL-4 production involves extended demethylation of the IL-4 gene. Immunity (2002) 16:649–60. 10.1016/S1074-7613(02)00314-X 12049717

[101] Cosmi L Liotta F Annunziato F. Th17 regulating lower airway disease. Curr Opin Allergy Clin Immunol. (2016) 16:1–6. 10.1097/ACI.0000000000000227 26600259

[102] Watanabe Y Onodera A Kanai U Ichikawa T Obata-Ninomiya K Wada T. Trithorax complex component Menin controls differentiation and maintenance of T helper 17 cells. Proc Natl Acad Sci USA. (2014) 111:12829–34. 10.1073/pnas.1321245111 25136117 PMC4156753

[103] Yamada T Kanoh M Nabe S Yasuoka T Suzuki J Matsumoto A. Menin plays a critical role in the regulation of the antigen-specific CD8+ T cell response upon listeria infection. J Immunol. (2016) 197:4079–89. 10.4049/jimmunol.1502295 27798149

[104] Iinuma T Okamoto Y Yamamoto H Inamine-Sasaki A Ohki Y Sakurai T. Interleukin-25 and mucosal T cells in noneosinophilic and eosinophilic chronic rhinosinusitis. Ann Allergy Asthma Immunol. (2015) 114:289–98. 10.1016/j.anai.2015.01.013 25704964

[105] Mitson-Salazar A Yin Y Wansley DL Young M Bolan H Arceo S. Hematopoietic prostaglandin D synthase defines a proeosinophilic pathogenic effector human T(H)2 cell subpopulation with enhanced function. J Allergy Clin Immunol. (2016) 137:907–18.e9. 10.1016/j.jaci.2015.08.007 26431580

[106] Wambre E Bajzik V DeLong JH O'Brien K Nguyen Q-A Speake C. A phenotypically and functionally distinct human TH2 cell subpopulation is associated with allergic disorders. Sci Transl Med. (2017) 9:eaam9171. 10.1126/scitranslmed.aam9171 28768806 PMC5987220

[107] Martinez RJ Evavold BD. Lower affinity T cells are critical components and active participants of the immune response. Front Immunol. (2015) 6:468. 10.3389/fimmu.2015.00468 26441973 PMC4564719

[108] Lauvau G Goriely S. Memory CD8+ T cells: orchestrators and key players of innate immunity? PLoS Pathog. (2016) 12:e1005722. 10.1371/journal.ppat.1005722 27584152 PMC5008753

[109] Schenkel JM Masopust D. Tissue-resident memory T cells. Immunity (2014) 41:886–97. 10.1016/j.immuni.2014.12.007 25526304 PMC4276131

[110] Russ BE Prier JE Rao S Turner SJ. T cell immunity as a tool for studying epigenetic regulation of cellular differentiation. Front Genet. (2013) 4:218. 10.3389/fgene.2013.00218 24273551 PMC3824109

